# DNA repair and recovery of RNA synthesis following exposure to ultraviolet light are delayed in long genes

**DOI:** 10.1093/nar/gkv148

**Published:** 2015-02-26

**Authors:** Leonardo C. Andrade-Lima, Artur Veloso, Michelle T. Paulsen, Carlos F.M. Menck, Mats Ljungman

**Affiliations:** 1 Department of Radiation Oncology and Translational Oncology Program, University of Michigan, Ann Arbor, MI, USA; 2 Department of Microbiology, Biomedical Sciences Institute, University of São Paulo, São Paulo, Brazil; 3 Department of Computational Medicine and Bioinformatics, University of Michigan, Ann Arbor, MI, USA; 4 Department of Environmental Health Sciences, University of Michigan, Ann Arbor, MI, USA

## Abstract

The kinetics of DNA repair and RNA synthesis recovery in human cells following UV-irradiation were assessed using nascent RNA Bru-seq and quantitative long PCR. It was found that UV light inhibited transcription elongation and that recovery of RNA synthesis occurred as a wave in the 5′-3′ direction with slow recovery and TC-NER at the 3′ end of long genes. RNA synthesis resumed fully at the 3′-end of genes after a 24 h recovery in wild-type fibroblasts, but not in cells deficient in transcription-coupled nucleotide excision repair (TC-NER) or global genomic NER (GG-NER). Different transcription recovery profiles were found for individual genes but these differences did not fully correlate to differences in DNA repair of these genes. Our study gives the first genome-wide view of how UV-induced lesions affect transcription and how the recovery of RNA synthesis of large genes are particularly delayed by the apparent lack of resumption of transcription by arrested polymerases.

## INTRODUCTION

Ultraviolet light (UV) from sunlight has through evolutionary time challenged all living organisms by damaging DNA. UVC light (254 nm) induces cyclobutane pyrimidine dimers (CPD) that effectively block elongating RNA polymerase II complexes (1,2). If transcription does not resume in a timely manner, cells may undergo apoptosis within 72 h (3–5). The UV-induced cell death occurs preferentially during S-phase presumably because of conflicts between replication machineries and blocked RNA polymerase complexes (6). It has been shown that blocked RNA polymerases recruit nucleotide excision repair factors in a CSA- and CSB-mediated manner allowing for a preferential repair of active genes (7) in a strand-specific manner (8). This form of repair, transcription-coupled nucleotide excision repair (TC-NER), has been assessed in mammalian genes including *DHFR, JUN, MYC* and *CDC2* (9–11) and *RBP2, URA3, MFA2 and GAL1–10* in yeast (12–14). Based on these results from a limited number of genes, it has been assumed that TC-NER operates similarly on all transcribing genes in the genome.

The human genome harbors approximately 23 000 genes each of which has its own unique chromatin structure shaped by histone modifications and DNA methylation. These epigenetic modifications dictate both the initiation and elongation rates of transcription (15). Whether TC-NER and global genomic NER (GG-NER) are affected by different epigenetic states and/or different initiation and elongation rates have not been assessed on a genome-wide scale. In addition to repair, recovery of RNA synthesis following repair may be influenced by the epigenetic environment. Interestingly, it has been shown that the recovery of RNA synthesis from the *DHFR* gene in CHO cells occurs faster than the removal of pyrimidine dimers from the transcribed strand (16). While some RNA polymerase complexes may be able to bypass lesions prior to their complete removal, perhaps after some initial modification of the damaged DNA, others are subjected to ubiquitylation and degradation (17–19). This ubiquitylation and degradation of the largest subunit of RNA polymerase II is thought to promote the removal of RNA polymerase complexes stalled at UV-induced DNA lesions and this degradation is defective in Cockayne's syndrome cells (19). If stalled, the RNA polymerases will shield the damage and therefore they need to be removed to allow access for repair factors. Subsequently, if RNA polymerases are ubiquitylated and removed, transcription would have to start over from the beginning of genes by new initiation. We recently found that RNA synthesis following release from camptothecin-induced inhibition of DNA topoisomerase I, recovers in a 5′ to 3′ direction (20). No recovery was observed in the middle or end of large genes suggesting that RNA polymerases blocked at sites of trapped DNA topoisomerases are not able to resume transcription even after the blocked DNA topoisomerases disengage or are removed from the DNA.

To explore the effects of UV-induced DNA damage and repair on transcription in human fibroblasts genome-wide, we used the newly developed Bru-seq technique (21,22). Bru-seq is based on metabolic labeling of nascent RNA using bromouridine (Bru) followed by deep sequencing of the immunoprecipitated nascent Bru-RNA. We found that UV light-induced DNA lesions inhibited elongation, but showed only limited effects on initiation of transcription. As cells were given time to repair the damage, the recovery was very slow in the 3′-end of large genes. Using quantitative long polymerase chain reaction (qPCR) we also found that UV-induced lesions were removed slower from 3′-ends of large genes than from 5′-ends. TC-NER-deficient CS-B cells showed a severely deficient recovery of RNA synthesis throughout genes after UV-irradiation, while XP-C cells, deficient in GG-NER, showed slower recovery at the 3′-end of large genes compared to wild-type cells. Surprisingly, individual genes in normal cells showed significant variation in RNA synthesis recovery that did not always correlate to repair efficiencies of these genes. This is the first genome-wide assessment of transcription recovery after UV-irradiation bringing new insights into the cellular response to DNA damage.

## MATERIALS AND METHODS

### Cell lines and cell culture

The following cell lines from primary human fibroblasts, obtained from the Coriell Repository were used in the project:

HF1—wild-type human fibroblasts obtained from foreskin and immortalized by hTERT, (kindly provided by Dr. Mary Davis, Department of Radiation Oncology, University of Michigan, USA).

XP67TMA (GM14867)—primary skin fibroblasts from a 7-year old male with homozygous mutations in *XPC* (obtained from Coriell Repository).

CS1AN (GM00739)—primary skin fibroblast from 3-year old female with compound heterozygote mutations in *CSB* (obtained from Coriell Repository).

All cell lines were grown as monolayers in Minimal Essential Medium (MEM) supplied with 10% fetal bovine serum and antibiotics (Invitrogen) and maintained at 37°C in a humidified 5% CO_2_ atmosphere.

### UV irradiation and Bru-seq

Cells were washed in phosphate buffered saline (PBS) and irradiated in 100 μl PBS on 100 mm plates with a UVC lamp producing 1 J/m^2^/s. Cells were then incubated in conditioned media for different periods of time (0, 6, 24h) before being incubated with 2 mM bromouridine (Bru) at 37°C for a 30 min. The cells were then lysed in TRIzol reagent (Invitrogen) and Bru-containing RNA isolated as previously described (21,22). cDNA libraries were made from the Bru-labeled RNA using the Illumina TruSeq library kit and sequenced using Illumina HiSeq sequencers at the University of Michigan DNA Sequencing Core. The sequencing and read mapping was carried out as previously described (21,22).

### DNA isolation and qPCR for DNA damage analysis

DNA was isolated with DNeasy Blood and Tissue kit (Qiagen) as described by the manufacturer and quantitated with Quant-iT dsDNA Picogreen kit (Invitrogen) using Fluorometer Polarstar Optima from BMG LABTECH emission filter at 520 nm. The qPCR was performed as previously described (23). In short, each sample was diluted to 3 ng/μl and 15 ng DNA was used for 40 μl qPCR reaction using TaKaRa LA PCR (TaKaRA Bio Group, Japan), initiated with a 85°C hot start addition of DNA polymerase (1–94°C for 3 min; 2–29 to 31 cycles at 94°C for 30 s plus 69°C for 9 min; 3–4°C). PCR primers and number of cycles used are described in Supplementary Table S1. To ensure quantitative amplification, a 50% DNA control (7.5 ng) was included in each sample set. To estimate potential DNA contaminations, a blank 1x TE sample with no DNA template, was included in each sample set. PCR products were run on 0.7% agarose gel to inspect size amplification and products were quantitated using Quant-iT dsDNA Picogreen kit (Invitrogen). The ratio of corrected values of fluorescence (blank sample subtracted) and irradiated samples divided by non-irradiated sample were calculated. Then we calculated the negative natural logarithm (-ln) of this ratio to determine the frequency of lesions per fragment based on Poisson distribution, assuming that DNA damage is randomly distributed across the genome. For strand-specific DNA damage analysis, we divided the remaining DNA lesions by 2 (sense and antisense) and subtracted the number of DNA lesions removed in non-transcribed regions (intergenic region and *LECT1*) in order to normalize and determine TC-NER in the transcribed strands of the tested genes. Two-way analysis of variance (two-way ANOVA) was utilized for statistical analysis, with *P*-values corresponding to <0.05 (*) assigned as significant.

### Data access

All the primary sequencing data files used in this study have been deposited in NCBI's Gene Expression Omnibus (GEO) with the accession number GSE65985.

## RESULTS

### Exposure to UVC light inhibits elongation of transcription

Assessments of the effects of UV-irradiation on transcription using total, steady-state RNA is confounded by the contributions of both RNA synthesis and degradation to the analyses. Thus, any transcriptional changes induced by UV-light will only be manifested in the steady-state RNA pool after a delay when they can be detected in the sea of previously made RNA. A more direct way to assess transcriptional effects of UV-irradiation is to assess changes in the rates of nascent RNA synthesis. Here we used the Bru-seq technique recently developed in our lab (21,22) to assess the genome-wide effects of UVC-irradiation on transcription in human fibroblasts. This technique is based on the metabolic labeling of nascent RNA in cultured dividing cells with bromouridine (Bru), followed by specific isolation of Bru-labeled nascent RNA from total RNA using anti-BrdU antibodies conjugated to magnetic beads. The isolated nascent RNA is then converted into cDNA libraries and deep sequenced. The sequenced reads are mapped to the human reference sequence and analyzed as previously described (21,22).

**Figure 1.**
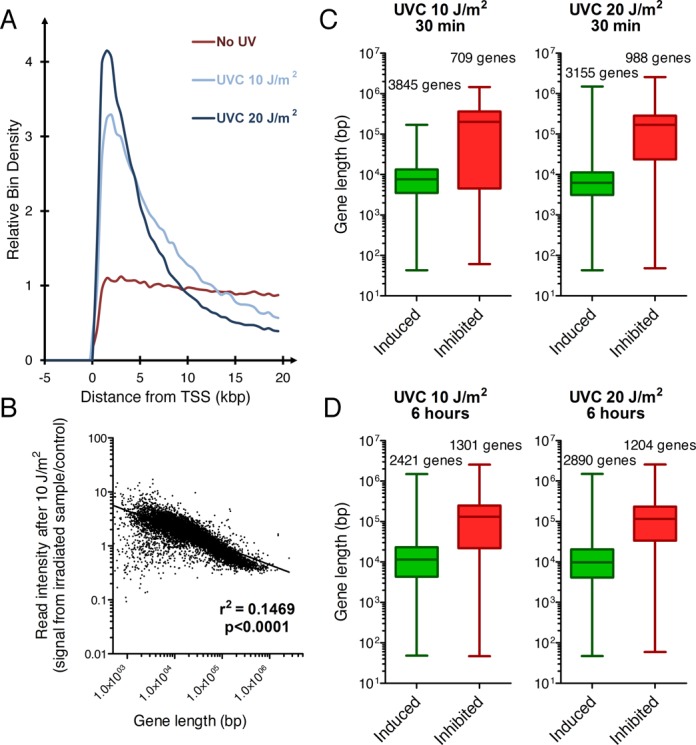
Irradiation with UVC light preferentially inhibits elongation with little effects on initiation of transcription. (**A**) Aggregate graph showing a UV dose-dependent reduction of reads in the bodies of genes and an enhancement of reads at the 5′-end of 988 genes longer than 20 kbp. Human fibroblasts were irradiated with UVC light and incubated with Bru for 30 min to label the nascent RNA and the nascent transcription reads are aligned from their transcription start site (TSS). (**B**) UV-mediated reduction in RNA synthesis is proportional to gene size. Ratio of Bru-seq signal (RPKM) of individual genes in UV-irradiated over control cells as a function of gene size. (**C**) The median length of genes showing relative induction or inhibition at least 2-fold directly after UV-irradiation (30 min Bru-labeling) or (**D**) following a 6-h recovery period. The gene maps are from RefSeq gene annotation (UCSC genome browser).

Here we applied Bru-seq to explore the genome-wide effects of UVC irradiation on nascent RNA synthesis in HF1 human fibroblasts. In Figure 1A, the data for genes larger than 20 kb long have been aligned from their transcription start sites (TSSs) and expressed as reads per thousand base pairs per million reads (RPKM). As can be seen, transcription reads from un-irradiated cells are evenly distributed from the TSSs and into the bodies of these genes (red trace). Exposure of the cells to 10 J/m^2^ (blue trace) or 20 J/m^2^ of UVC irradiation (black trace) altered the distribution of transcription output in a dose-dependent manner with enhancement of reads at the beginning of genes while loss of reads in the bodies of genes. Since Bru-seq data represent the distribution of reads rather than absolute expression values, when RNA synthesis is reduced in the body of large genes, sequencing reads must accumulate elsewhere. Since transcription initiation is less affected than elongation globally by UV light, reads accumulate at the 5′-end of genes. Among the 22 984 human genes (RefSeq annotation), the mean expression in the human fibroblasts HF1 was 1.09 reads per thousand base pairs per million reads (RPKM), with 13251 genes that had expression lower than 0.3 RPKM, while the median was 0.15 RPKM due to the large number of genes without any expression. Since UV-induced DNA lesions effectively block transcription elongation, large genes are expected to be inhibited more than are smaller genes since they represent larger targets of inactivation. Indeed, the phenomenon that transcription inhibition is proportional to gene size was originally utilized to estimate gene sizes in viruses and cells (24). As expected, irradiation of human fibroblasts with 10 J/m^2^ of UVC lead to a negative correlation between the expected read intensity and gene length (Figure 1B). The median length of genes with more than 2-fold decreased relative RNA synthesis was 201.77 kbp after 10 J/m^2^ of UVC light, while the median gene length with more than 2-fold increased relative transcription was 7.67 kbp (Figure 1C, upper panel). Following irradiation with 20 J/m^2^, the median length of genes with increased relative transcription decreased to 6.22 kbp, while the genes with decreased relative transcription decreased to 169.3 kbp. After 6 h post irradiation, a limited recovery was observed with an increase in the average gene size of genes with 2-fold increase in relative RNA synthesis while a decrease in the average gene size for genes with a 2-fold decreased relative RNA synthesis (Figure 1D). These results show that UV-irradiation preferentially inhibits transcription of large genes and thus, there is a switch in the relative RNA synthesis favoring short genes.

### Global genomic nucleotide excision repair (GG-NER) contributes to transcription recovery following UV light in larger genes

**Figure 2.**
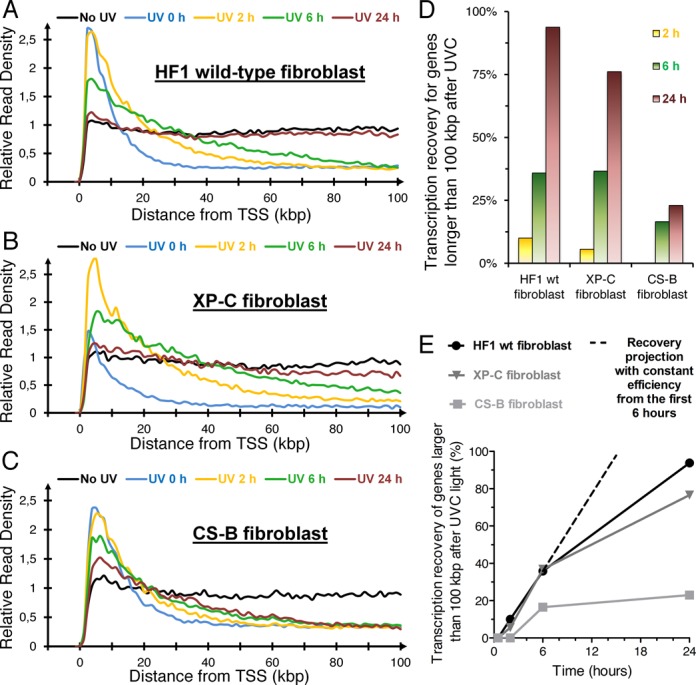
Transcription recovery following 10 J/m^2^ of UVC light of long genes (> 100 kbp) in wild-type human primary fibroblasts or fibroblast deficient in nucleotide excision repair. After UVC light irradiation and recovery, a 30 min Bru labeling was performed before RNA isolation. (**A**) Aggregate graph of wild-type TC-NER and GG-NER-proficient fibroblast HF1 of 292 genes longer than 100 kb with an average expression above 0.5 RPKM. (**B**) Aggregate graph of XP67TMA GG-NER-deficient fibroblasts (mutation of *XPC*) of 296 genes longer than 100 kb with an average expression above 0.5 RPKM. (**C**) Aggregate graph of CS1AN TC-NER-deficient fibroblasts (mutation in *CSB*) of 289 genes longer than 100 kb with an average expression above 0.5 RPKM. (**D**) Comparison of the percent transcription recovery following UVC light between the different cell lines in A–C. (**E**) Percent recovery of RNA synthesis plotted as a function of time.

Wild-type human HF1 fibroblasts showed progressive RNA synthesis recovery following UV-irradiation (Figure 2A). After 2 h (yellow) and 6 h (green) of repair, the distribution of RNA synthesis signal changed in the beginning and middle of large genes while no change of RNA synthesis was observed at the 3′-end of large genes. After a 24-h repair period (red), the recovery of RNA synthesis was complete even at the 3′-ends of long genes. This was different for GG-NER-deficient XPC fibroblasts where we observed a statistically significant diminished recovery in the 3′-end of long genes at 24 h (*P* < 0.0001) (Figure 2B). As expected, the recovery of transcription was severely diminished after UV-irradiation in TC-NER-deficient CSB cells with only a modest recovery observed in the beginning of the genes (Figure 2C). These results indicate that the recovery of RNA synthesis is slower at the 3′end of large genes and that GG-NER contributes to the recovery of RNA synthesis at the 3′-ends of large genes.

To compare the recovery of RNA synthesis after UV-irradiation between different cell lines, we calculated the percentage of transcription recovery for each cell line at different times after UV-irradiation (Figure 2D). It can be seen that the CS-B cells lacking TC-NER have a substantially retarded recovery of RNA synthesis both when examining RNA synthesis at 6 and 24 h after exposure to UV light. The XP-C cells, on the other hand, show proficient recovery at 6 h but a reduced recovery at 24 h post-irradiation compared to normal fibroblasts but significantly more recovery than for the CS-B cells. The rate of recovery is high during the first 6 h and then the rate is leveling off (Figure 2E). If the same efficiency was maintained, XP -C cells should be fully recovered RNA synthesis after 15 h, however we observe only 76% recovery by 24 h. Our data suggest that efficient recovery of RNA synthesis at early time points require functional TC-NER while at later time points, GG-NER plays a role presumably by contributing to removal of UV-lesions at the 3′-ends of long genes, where TC-NER would not yet have reached.

### Transcription recovery following UVC light is independent of the level of gene expression

**Figure 3.**
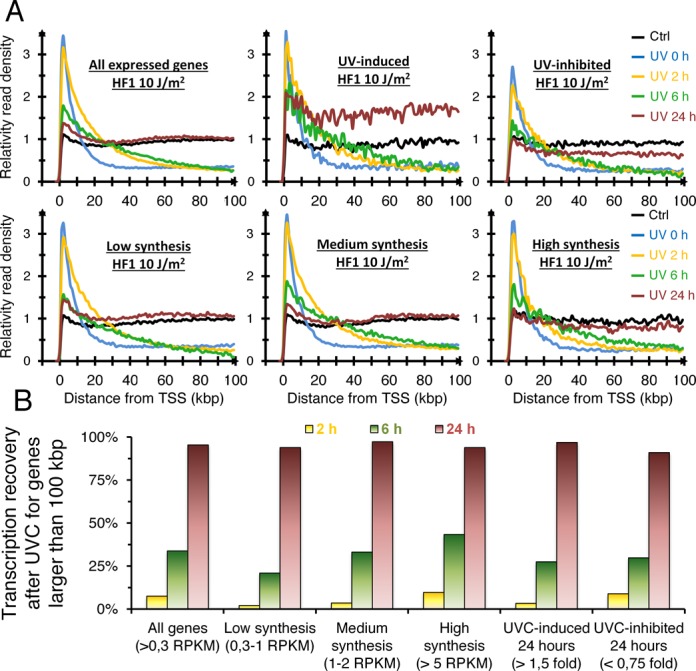
Transcription recovery of genes after UV-irradiation occurs to similar rates regardless of their expression level or whether they are induced or repressed by UV light. (**A**) Aggregate graphs of long genes (>100 kbp) with different patterns of gene expression at different times following exposure to 10 J/m^2^: All genes (1496 genes, synthesis >0.3 RPKM); Low expression (720 genes, 0.3 RPKM <synthesis <1 RPKM in non-irradiated control); Medium expression (485 genes, 1 RPKM <synthesis <2 RPKM in non-irradiated control); High expression (57 genes, synthesis >5 RPKM in non-irradiated control); UVC-induced 24 h post irradiation (92 genes with ratio >1.5 fold); UVC-inhibited 24 h post irradiation (197 genes with ratio <0.75 fold). (**B**) Percent transcription recovery following 10 J/m^2^ of UVC light for the samples shown in A).

It is plausible that highly transcribed genes may clear RNA polymerase blockage more rapidly because of more frequent encounters of lesions by the RNA polymerases. To test this hypothesis, we arranged genes larger than 100 kbp into three groups according to the transcription rates observed in un-irradiated cells: (1) high expression (RNA synthesis > 5 RPKM); (2) medium expression (1 < RNA synthesis < 2 RPKM); (3) low expression (0.3 < RNA synthesis < 1 RPKM). We also obtained the list of genes that showed >1.5-fold relative induction of transcription at 24 h post-irradiation and (5) a list of genes with a >1.5-fold relative decrease of transcription signal at 24 h. We found that the RNA synthesis recovery profiles of highly expressed genes did not differ significantly from moderately or lowly expressed genes (Figure 3A and B). Similarly, genes with relative higher or lower RNA synthesis showed similar efficiencies of transcription recovery following UV exposure (Figure 3B). To compare RNA synthesis recovery between genes with distinct expression, we analyzed distribution of sequencing read throughout the genes. Without DNA damage, genes present an even distribution of signal, but after UV-irradiation the reads are reduced in the bodies of genes with the subsequent increase of reads near TSSs. A 100% recovery would mean that the transcription signal throughout the gene has returned back to an even distribution. These results indicate that the level of transcription of a gene does not influence the recovery of RNA synthesis following DNA damage.

### Variable transcription recovery in individual genes

**Figure 4.**
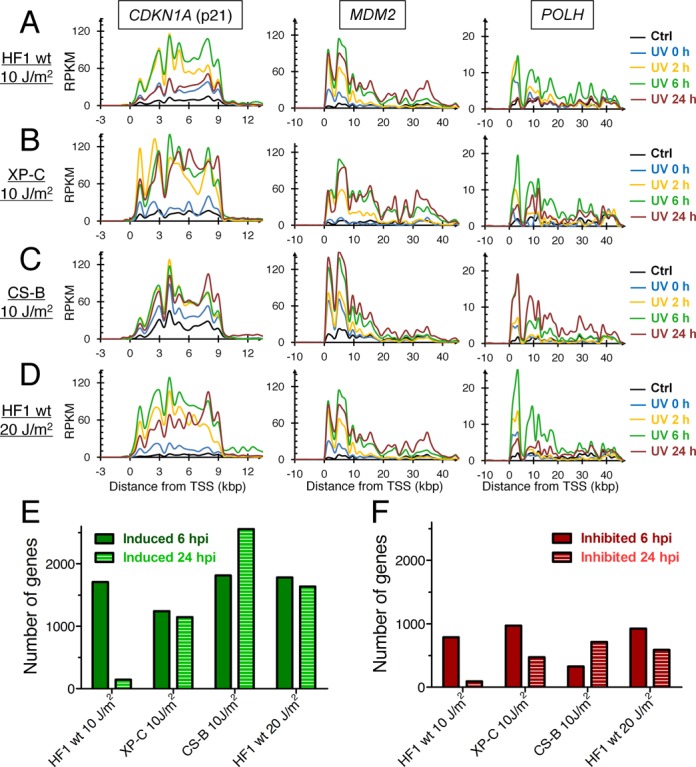
The UV-induced RNA synthesis of the p53-inducible *CDKN1A, MDM2* and *POLH* genes returns to basal levels 24 h after exposure to 10 J/m^2^ in wild-type fibroblasts HF1, but not in NER-deficient cells lines or in wild-type cells irradiated with 20 J/m^2^. Direction of transcription is from left to right. (**A**) HF1, (**B**) XP-C, (**C**) CS-B (**D**) HF1, irradiated with 20 J/m^2^. (**E**) Number of genes 2-fold induced 6 or 24 h post irradiation. (**F**) Number of genes 2-fold inhibited 6 or 24 h post irradiation.

We next evaluated RNA synthesis inhibition and recovery in individual genes following UV-irradiation. We observed that short genes such as *CDKN1A* (10.8 kbp) were not inhibited by 10 J/m^2^ of UVC light as no decrease was found in RNA synthesis signal towards the 3′-end of the gene (Figure 4A). With this dose of UVC light, we expect the generation of on average 1 CPD per 7 kbp (double stranded DNA) or 1 CPD per 14 kb on the transcribed strand (16). Since the induction of different CPDs occur independent of each other, we can use a Poisson distribution to model the probability of lesion frequency in any given gene. Applying the Poisson distribution to estimate CPD frequencies in the CDKN1A gene, we expect that about 45% of the cells will have a lesion-free CDKN1A gene; 36% of cells would have one lesion; 14% of cells will have two lesions and the remaining 5% of cells would be expected to have 3 or more lesions. Because the CDKN1A gene is a transcriptional target of p53 (25), we observed increased induction of transcription of the CDKN1A gene with maximal expression at the 6-h time point after irradiation with synthesis restored to basal level by 24 h post-irradiation (Figure 4A). For larger p53-inducible genes, such as *MDM2, POLH* (Figure 4A), *DRAM1* and SESN1 (Supplementary Figure S1), the distribution of reads were shifted to the 5′-ends immediately after UV-irradiation as we observed in the aggregate view of long genes (Figure 1). For *SESN1*, two promoters were utilized for transcription where only the 3′-proximal promoter was p53-inducible (Supplementary Figure S1A). Similarly to *CDKN1A*, the *MDM2, POLH, DRAM1* and *SESN1* genes showed maximal RNA synthesis 6 h after UV-irradiation with transcription returning to basal levels by 24 h. In GG-NER-deficient XP-C cells, we observed a sustained induction of transcription from the *CDKN1A* and *MDM2* genes, but this was not evident for the *POLH* and *DRAM1* genes (Figure 4B, Supplementary Figure S1B). In contrast, CS-B cells showed sustained induction of all five genes after UV-irradiation (Figure 4C and Supplementary Figure S1C). Finally, cells exposed to 20 J/m^2^ showed sustained induction of *CDKN1A* and *MDM2* up to 24 h post-irradiation while *POLH, DRAM1* and *SESN1* did not show this as strongly (Figure 4D, Supplementary Figure S1D). A similar response was observed when we analyzed all the genes that were induced at least 2-fold (Figure 4E) or inhibited at least 2-fold by UV-irradiation (Figure 4F). These results show that the induction and repression of individual genes after UV light exposure is prolonged in DNA-repair deficient cells.

### Differential repair of 5′ and 3′-ends of large genes

**Figure 5.**
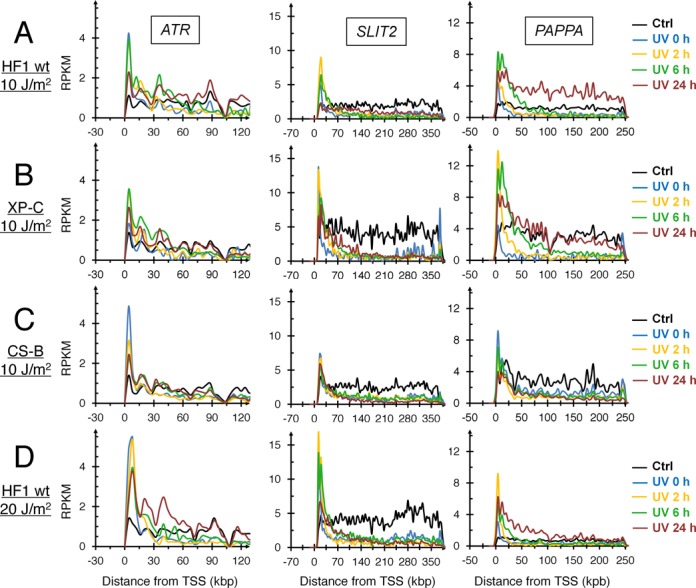
Transcription recovery in the *ATR, SLIT2* and *PAPPA* genes following 10 J/m^2^ of UVC light. Direction of transcription is from left to right. (**A**) HF1, (**B**) XP-C, (**C**) CS-B, (**D**) HF1, 20 J/m^2^. Note that the *SLIT2* gene shows no appreciable RNA synthesis recovery even after 24 h of recovery. The *PAPPA* gene is a known p53-inducble gene.

The recovery of RNA synthesis was found to be faster in the 5′-end than in the 3′-end of large genes in wild-type cells, which may reflect either a faster rate of CPD removal or a more frequent traversal of RNA polymerases stimulating TC-NER. Examining individual genes, we surprisingly found that the recovery of RNA synthesis differed dramatically between genes. For example, the *ATR* and *PAPPA* genes showed full recovery of RNA synthesis within 24 h while no recovery was observed for the *SLIT2* gene despite being expressed at a similar level as the other two genes (Figure 5). These results suggest that recovery of RNA synthesis following UV irradiation is not correlated to the level of expression of the gene but may be linked to either rates of DNA repair or gene-specific re-initiation of transcription following the completion of repair. Indeed, the recovery of RNA synthesis in GG-NER-deficient XP-C cells was slower at the 3′end of large genes than in wild-type cells suggesting that GG-NER contributes to the recovery of RNA synthesis at the 3′-end of genes.

To investigate whether the different rates of recovery of RNA synthesis in the 5′ and 3′-ends of genes and between different genes correlate to different rates of DNA repair, we used qPCR to estimate lesion frequencies at different locations within genes (23). In this assay, genomic DNA is collected from UV-irradiated cells after different periods of time after UV-irradiation and PCR primers were selected to allow amplifications of 10 kbp segments of DNA in the respective genes. The rationale for this approach is that amplification is inversely proportional to the frequency of segments containing transcription-blocking CPDs or UV 6–4 photoproducts. As repair proceeds, more amplification of the segment will be expected. We used this assay to compare the rates of removal of UV lesions from different parts of genes, and among genes with different RNA synthesis recovery profiles. Furthermore, these experiments were performed on GG-NER-defective XP-C fibroblasts so that any repair we observed had to be due to TC-NER and not GG-NER. The data were normalized with the non-transcribed regions so that we could determine DNA repair on the sense strand.

**Figure 6.**
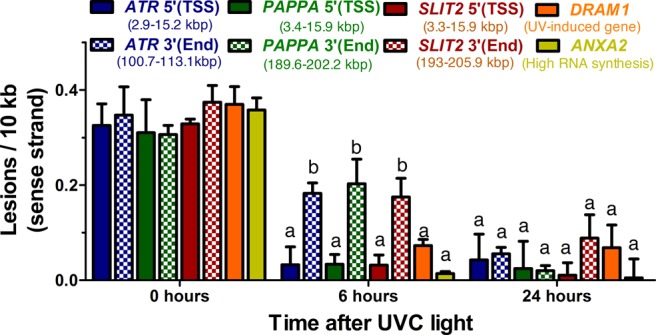
DNA lesion removal from the sense strand of the 5′-ends (TSS) and 3′-ends (END) of the genes *ATR, PAPPA, SLIT2, ANXA2* and *DRAM1*. TC-NER deficient XP-C cells were irradiated with 10 J/m^2^ of UVC light and lesion removal estimated using qPCR. Note that lesions are repaired slower from the 3′-end compared to the 5′-end of long genes and similarly in the SLIT2 gene that showed no RNA synthesis recovery. Remaining DNA lesions are expressed as lesions/10 kb of the sense strand as calculated assuming a Poisson distribution (see Materials and Methods). The values represents the mean of quadruplicate samples from two independent biological experiments with bars representing the standard deviation. Sense strand normalization was related to DNA repair obtained from non-transcribed genes. Statistics with Two-way ANOVA: ‘a’ is significantly different from ‘b’.

Using PCR primers specific for the 5′ and 3′ ends for *ATR, PAPPA* and *SLIT2* genes as well as primers for a non-transcribed intergenic region near the ATR gene on chromosome 3, we found faster removal of CPDs and other photoproducts near transcription start site (5′-end) compared to the 3′-end in all three genes while no repair was found in the non-transcribed region adjacent to the ATR gene (Figure 6). Strikingly, there were no statistical differences in the efficiencies of lesion removal from the 3′-end of these three genes despite their marked differences in transcription recovery (Figure 6). These findings implicate that the removal of CPDs and other UV-lesions by TC-NER is faster at the 5′-ends compared to the 3′-ends of genes and that DNA repair is not always accompanied by recovery of RNA synthesis but may require additional gene-specific factors for induction. To further investigate the relationship between efficiency of DNA repair and the level of RNA synthesis recovery in XP-C cells we tested three additional genes: *ANXA2* (high basal RNA synthesis); *DRAM1* (UV-induced) (Supplementary Figure S1B) and *LECT1* (not expressed). While *LECT1* showed almost no DNA repair (Supplementary Figure S6), *DRAM1* and *ANXA1* showed similar DNA repair rates as the other three genes (Figure 6). Surprisingly, the lack of recovery of RNA synthesis from the *SLIT2* gene was not caused by the absence of repair since it showed similar repair rates as for genes with faster RNA synthesis recovery, such as ATR and PAPPA (Figures 5 and 6). These results support the idea that high transcription rates do not necessarily result in faster TC-NER and that TC-NER does not alone explain the differences in the profile of RNA synthesis recovery in different genes.

### Genes with induced relative RNA synthesis following UV-irradiation

In Figure 1, we showed that gene size largely determines the effects of UV-irradiation on relative rates of RNA synthesis. Here we performed DAVID gene enrichment analysis (26) of the gene sets displayed in Figure 4 E and F, with more than 2-fold altered rates of relative transcription following UV-irradiation. Wild-type fibroblasts irradiated with 10 J/m^2^ exhibited 1709 genes with more than 2-fold increased relative transcription at 6 h (Figure 4E). These genes showed enrichment for KEGG pathways such as ‘ribosome’, ‘p53 signaling’, ‘cell cycle’, ‘RNA degradation’, ‘nucleotide excision repair’ and ‘apoptosis’ (Supplementary Figure S2). Among the genes showing the highest increase in relative RNA synthesis were the p53-inducible genes *BTG2, CDKN1A, PCNA, DDB2* and *FAS* (Supplementary Table S1). After 24 h post-irradiation, the 69 genes that showed more than 2-fold elevated relative expression where enriched for pathways such as ‘cell cycle’ and ‘apoptosis’.

In GG-NER-deficient XP-C and TC-NER-deficient CS-B cells, the list of genes showing 2-fold induced relative transcription was very similar to that observed in wild-type fibroblasts (Figure 4E), with 1243 and 1812 genes showing elevated relative transcription, respectively. However, at 24 h after UV irradiation, 1144 genes showed increased relative transcription in XP-C cells, whereas CS-B cells showed increased relative transcription of 2556 genes. DAVID gene enrichment analyses showed in addition enrichment for cellular functions such as ‘regulation of autophagy’ and ‘base excision repair’. Wild-type fibroblasts irradiated with 20 J/m^2^, showed 1634 genes with at least a 2-fold increased relative rate of transcription at 24 h following irradiation and this gene set showed additional enrichment for ‘lysosome’, ‘gluthatione metabolism’, ‘chemokine signaling’ and ‘apoptosis’ (Supplementary Figure S5B).

### Genes with repressed relative RNA synthesis following UV-irradiation

Large genes are preferentially targeted for inactivation of UV irradiation due to the inhibitory effect of UV-induced DNA lesions on transcription elongation (Figure 1B). Here we found that UV-irradiation inhibited the relative transcription of 787 genes 6 h after exposure to 10 J/m^2^. DAVID analyses of this gene set found enrichment for ‘focal adhesion’, ‘calcium signaling’, ‘regulation of actin cytoskeleton’ and ‘tight junctions’ (Supplementary Figure S2C). Twenty-four hours after UV-irradiation, 91 genes showed 2-fold reduced relative RNA synthesis, and these genes only showed enrichment for encoded proteins involved in ‘focal adhesion’ and ‘regulation of actin cytoskeleton’ (Supplementary Figure S2D). For XP-C cells, UV-irradiation generated a similar profile of repressed genes as for wild-type cells at 6 h while many more genes (971) showed reduced relative transcription at 24 h (Supplementary Figure S3D). CS- B cells had 326 genes inhibited at least 2-fold at 6 h post UV-irradiation and this number increased to 713 after 24 h post-irradiation and these additional genes showed enrichment for ‘regulation of cell cycle’ (Supplementary Figure S4D).

## DISCUSSION

It is well known that UV-irradiation effectively inhibits the synthesis of nascent RNA by blocking transcription elongation at sites of CPDs and 6–4 photoproducts (27). These lesions are preferentially removed from the transcribed strands of actively transcribing genes by TC-NER (8,28). Whether TC-NER works on all protein-coding and non-coding genes transcribed by RNA polymerase II is not known. To remove the blocking lesions and resume transcription, cells have to first remove the blocked RNA polymerases from the damaged sites to allow access for NER factors (29). It is not clear whether cells can reuse the blocked RNA polymerases to resume RNA synthesis or whether these polymerases are consumed and eliminated by ubiquitylation and degradation (18,19).

To assess inhibition and recovery of RNA synthesis following UV-irradiation in individual genes genome-wide we used the nascent RNA Bru-seq technique. Our results show that irradiation of human fibroblasts with UVC inhibited elongation while initiation of transcription was in general not inhibited, which is in contrast to a previous study (30). The overall inhibition of the transcription signal from a gene was, as expected, found to be length-dependent. This lead to a significant redistribution of the reads within genes with reads highly enriched just downstream of transcription start sites while suppressed in the bodies of larger genes. This phenomenon explained in large parts gene expression results obtained shortly after UV exposure when short genes showed induced relative rates of RNA synthesis while large genes showed reduced rates due to the inclusion of long segments of suppressed transcription (Figure 1B). After different periods of recovery following UV-irradiation, we observed changes in the RNA synthesis profiles with rapid changes in the beginning and middle of the genes while a delayed recovery towards the 3′-ends of large genes. This slow recovery at the 3′-ends of large gene was further delayed in GG-NER-deficient XP-C cells and absent in TC-NER deficient CS-B cells (Figure 2). Furthermore, the rate of recovery of RNA synthesis was not correlated to the expression level of the gene, even though we cannot distinguish between cell cycle specific expression in these experiments. These results suggest that recovery of RNA synthesis following UV-irradiation occurs in a 5′ to 3′ direction, requires TC-NER, and is enhanced by GG-NER. Recent findings show that NER factors, such as XPC, may play roles in the transcription process in the absence of exogenous DNA damage (31). Interestingly, the rates of RNA synthesis recovery of individual genes were not dictated by the expression level of the genes themselves. Furthermore, the initial lack of recovery of transcription in the 3′ end of large genes would be consistent with a model suggesting that RNA polymerases blocked at lesions cannot be used to resume RNA synthesis even after the blocking lesions are removed.

**Figure 7.**
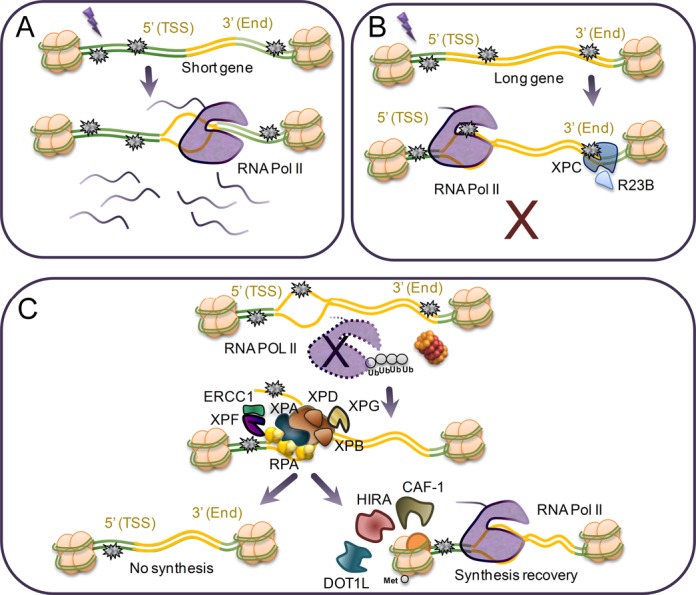
Model of transcription recovery following irradiation with UVC light. (**A**) Short genes are less likely to suffer DNA damage and thus transcription is less inhibited in these genes compared to larger genes. (**B**) The induction of DNA damage and the resulting inhibition of transcription elongation are proportional to gene length. GG-NER (XPC protein) contributes to the recovery of transcription in long genes by removal of DNA lesions in the 3′-end of these genes. (**C**) The arrested RNAPII needs to be backed up or removed from the site of the blocking DNA lesion to allow access to the NER factors. Since the recovery of RNA synthesis appears to be a process that occurs in a 5′-3′ direction, it is plausible that the blocked RNAPII is released or targeted for degradation. Removal of transcription-blocking lesions was faster in the 5′-ends of genes compared to the 3′-ends of large genes. RNA synthesis did not recover in certain genes (*SLIT2*) despite showing proficient removal of UV-lesions. Thus, factors such as HIRA, CAF-1 and DOT1L, may be needed for efficient re-start of RNA synthesis following repair of UV-induced damage.

Investigating RNA synthesis recovery in individual large genes in repair-proficient fibroblasts revealed a delayed recovery at the 3′-end (Figure 5A, Supplementary Figure S1A). This recovery was further delayed in XP-C cells and absent in CS-B cells (Figure 5B and C, Supplementary Figure S1B and C). Strikingly, genes such as *SLIT2*, showed virtually no recovery even in repair-proficient HF1 cells (Figure 5A). To test whether the slow recovery at the 3′-end of large genes and the poor recovery of RNA synthesis in *SLIT2* were due to slow rates of TC-NER we used qPCR with primers covering 5′ and 3′ ends of selected large genes in XP-C cells. While DNA damage induction was similar throughout the gene body, the removal of CPDs and other photoproducts was markedly faster near transcription start site (5′-end) compared to the 3′-end of active genes while no repair was observed in a non-transcribed gene or intergenic region (Figure 6). These results suggest that TC-NER operates in a 5′ to 3′ direction and that transcription recovery is linked to lesion removal. Surprisingly, the repair rate in the *SLIT2* gene was very similar to the other genes investigated although this gene showed no evidence of transcription recovery during the first 24 h following UV-irradiation. Thus, while lesion removal may be a prerequisite for cells to resume transcription, it does not seem to be sufficient. It is possible that re-start of transcription following the repair of UV-induced DNA lesions require specific factors that may operate in a gene-specific fashion. A factor that has been identified to play an important role in restarting transcription after UV repair is the histone chaperone HIRA (histone chaperone histone regulator A) (32). HIRA deposits H3.3 histone variants in transcribed regions of damaged chromatin prior to completion of DNA repair. CAF-1 (chromatin assembly factor 1) is another histone chaperone that deposits H3.1 variant histones into chromatin in response to DNA damage (33). Finally, the epigenetic regulator DOT1L, which specifically methylates H3K79, has been implicated in transcription re-start after UV-irradiation (34). It is possible that some genes, such as *SLIT2*, may not recruit these factors as efficiently after UV-irradiation and therefore they lack the ability to restart transcription even following removal of the blocking lesions (Figure 7).

Translesion RNA synthesis is a potential mechanism by which transcription can proceed with RNA synthesis on a damaged template (16). Studies in yeast have shown that translesion RNA synthesis occurs at a low frequency and that specific mutations of RNA polymerase at sites responsible for translesion RNA synthesis decreased cell survival following exposure to UVC light (35). However, we found no evidence for general translesion RNA synthesis in human fibroblasts, since we observed a permanent arrest of RNAPII for several hours following UV-irradiation. In order to make the UV lesions accessible to the NER proteins, the RNA polymerase has to either backtrack or be removed from the template. Although our Bru-seq data cannot distinguish between these different possibilities, we observed that transcription recovery of large genes (over 100 kbp) is not constant over time. RNA polymerases that were synthesizing RNA from the 3′-ends of genes at the time of UV-irradiation would be initially arrested at the 3′-ends of those genes. Why then do we not see recovery occurring at the 3′-ends of large genes until 24 h? Our data suggest that RNA polymerases arrested at sites of UV-induced lesions are targeted for removal and possibly degradation, requiring that all new RNA synthesis have to resume from the beginning of genes. In support of this hypothesis, we recently reported that transcription is forced to abort and re-start from the beginning of genes after human fibroblasts were transiently exposed to the DNA topoisomerase I inhibitor camptothecin (20). It is possible that the energy consumed by this abortive behavior of RNA polymerases may be offset by the advantage of temporal delay of expression of long gene under conditions of genotoxic stress.

UV light has challenged the integrity of DNA in all living organism throughout evolutionary history. The striking size differences among the nearly 23 000 genes in the human genome is the major determinant of the unique transcription pattern observed following UV-irradiation, where initial inhibition of transcription is proportional to the genomic size of the transcription unit. Have gene sizes been selected according to their functional role following UV-irradiation, so that genes beneficial to cells following UV exposure are small and compact while genes with no benefit or detrimental have been selected to be large? The pro-survival genes *MDM2, BCL2L1* and *PPMD1* are nearly 10 times longer than the pro-apoptotic genes *PUMA, BAX* and *BAK1* despite having similar mature mRNA size (36). This size difference will ensure that apoptosis will ensue following UV exposures exceeding a critical threshold. In this study we combined nascent RNA synthesis and DNA repair assessments to study the interplay between DNA repair and transcription following exposures to the prominent environmental mutagen UV light. Our study is the first of its kind to assess the genome-wide effects of UV-irradiation on RNA synthesis and revealed novel insights into the cellular responses to UV light.

## SUPPLEMENTARY DATA

Supplementary Data are available at NAR Online.

SUPPLEMENTARY DATA

## References

[1] Tornaletti S., Hanawalt P. (1999). Effect of DNA lesions on transcription elongation. Biochimie.

[2] Tornaletti S., Reines D., Hanawalt P.C. (1999). Structural characterization of RNA polymerase II complexes arrested by a cyclobutane pyrimidine dimer in the transcribed strand of template DNA. J. Biol. Chem..

[3] Ljungman M., Zhang F. (1996). Blockage of RNA polymerase as a possible trigger for u.v. light-induced apoptosis. Oncogene.

[4] McKay B., Ljungman M. (1999). Role for p53 in the recovery of transcription and protection against apoptosis induced by ultraviolet light. Neoplasia.

[5] Ljungman M., Lane D.P. (2004). Transcription - guarding the genome by sensing DNA damage. Nat. Rev. Cancer.

[6] McKay B., Becerril C., Spronck J., Ljungman M. (2002). Ultraviolet light-induced apoptosis is associated with S-phase in primary human fibroblasts. DNA Repair.

[7] Bohr V.A., Smith C.L., Okumoto D.S., Hanawalt P.C. (1985). DNA repair in an active gene: removal of pyrimidine dimers from the DHFR gene of CHO cells is much more efficient than in the genome overall. Cell.

[8] Mellon I., Spivak G., Hanawalt P.C. (1987). Selective removal of transcription-blocking DNA damage from the transcribed strand of the mammalian DHFR gene. Cell.

[9] An J., Yang T., Huang Y., Liu F., Sun J., Wang Y., Xu Q., Wu D., Zhou P. (2011). Strand-specific PCR of UV radiation-damaged genomic DNA revealed an essential role of DNA-PKcs in the transcription-coupled repair. BMC Biochem..

[10] Tommasi S., Oxyzoglou A.B., Pfeifer G.P. (2000). Cell cycle-independent removal of UV-induced pyrimidine dimers from the promoter and the transcription initiation domain of the human CDC2 gene. Nucleic Acids Res..

[11] Tu Y.Q., Tornaletti S., Pfeifer G.P. (1996). DNA repair domains within a human gene: selective repair of sequences near the transcription initiation site. EMBO J..

[12] Li S., Smerdon M.J. (2004). Dissecting transcription-coupled and global genomic repair in the chromatin of yeast GAL1–10 genes. J. Biol. Chem..

[13] Teng Y., Li S., Waters R., Reed S.H. (1997). Excision repair at the level of the nucleotide in the Saccharomyces cerevisiae MFA2 gene: mapping of where enhanced repair in the transcribed strand begins or ends and identification of only a partial rad16 requisite for repairing upstream control sequences. J. Mol. Biol..

[14] Tijsterman M., Verhage R.A., van de Putte P., Tasseron-de Jong J.G., Brouwer J. (1997). Transitions in the coupling of transcription and nucleotide excision repair within RNA polymerase II-transcribed genes of Saccharomyces cerevisiae. Proc. Natl. Acad. Sci. U.S.A..

[15] Veloso A., Kirkconnell K.S., Magnuson B., Biewen B., Paulsen M.T., Wilson T.E., Ljungman M. (2014). Rate of elongation by RNA polymerase II is associated with specific gene features and epigenetic modifications. Genome Res..

[16] Ljungman M. (1999). Recovery of RNA synthesis from the DHFR gene following UV-irradiation precedes the removal of photolesions from the transcribed strand. Carcinogenesis.

[17] Bregman D.B., Halaban R., Vangool A.J., Henning K.A., Friedberg E.C., Warren S.L. (1996). UV-induced ubiquitination of RNA polymerase II: A novel modification deficient in cockayne syndrome cells. Proc. Natl. Acad. Sci. U.S.A..

[18] Ratner J.N., Balasubramanian B., Corden J., Warren S.L., Bregman D.B. (1998). Ultraviolet radiation-induced ubiquitination and proteasomal degradation of the large subunit of RNA polymerase II - Implications for transcription-coupled DNA repair. J. Biol. Chem..

[19] McKay B.C., Chen F., Clarke S.T., Wiggin H.E., Harley L.M., Ljungman M. (2001). UV light-induced degradation of RNA polymerase II is dependent on the Cockayne's syndrome A and B proteins but not p53 or MLH1. Mutat. Res..

[20] Veloso A., Biewen B., Paulsen M.T., Berg N., Carmo de Andrade Lima L., Prasad J., Bedi K., Magnuson B., Wilson T.E., Ljungman M. (2013). Genome-wide transcriptional effects of the anti-cancer agent camptothecin. PloS One.

[21] Paulsen M.T., Veloso A., Prasad J., Bedi K., Ljungman E.A., Tsan Y.C., Chang C.W., Tarrier B., Washburn J.G., Lyons R. (2013). Coordinated regulation of synthesis and stability of RNA during the acute TNF-induced proinflammatory response. Proc. Natl. Acad. Sci. U.S.A..

[22] Paulsen M.T., Veloso A., Prasad J., Bedi K., Ljungman E.A., Magnuson B., Wilson T.E., Ljungman M. (2014). Use of Bru-Seq and BruChase-Seq for genome-wide assessment of the synthesis and stability of RNA. Methods.

[23] Furda A.M., Bess A.S., Meyer J.N., Van Houten B. (2012). Analysis of DNA damage and repair in nuclear and mitochondrial DNA of animal cells using quantitative PCR. Meth. Mol. Biol..

[24] Sauerbier W., Hercules K. (1978). Gene and transcription unit mapping by radiation effects. Ann. Rev. Genetics.

[25] Wei C.L., Wu Q., Vega V.B., Chiu K.P., Ng P., Zhang T., Shahab A., Yong H.C., Fu Y., Weng Z. (2006). A global map of p53 transcription-factor binding sites in the human genome. Cell.

[26] Huang da W., Sherman B.T., Lempicki R.A. (2009). Systematic and integrative analysis of large gene lists using DAVID bioinformatics resources. Nat. Protoc..

[27] Friedberg E., Walker G., Siede W., Wood R., Schultz R., Ellenberger T. (2006). DNA Repair and Mutagenesis.

[28] Hanawalt P.C., Spivak G. (2008). Transcription-coupled DNA repair: two decades of progress and surprises. Nat. Rev. Mol. Cell Biol..

[29] Brueckner F., Hennecke U., Carell T., Cramer P. (2007). CPD damage recognition by transcribing RNA polymerase II. Science.

[30] Rockx D.A., Mason R., van Hoffen A., Barton M.C., Citterio E., Bregman D.B., van Zeeland A.A., Vrieling H., Mullenders L.H. (2000). UV-induced inhibition of transcription involves repression of transcription initiation and phosphorylation of RNA polymerase II. Proc. Natl. Acad. Sci. U.S.A..

[31] Le May N., Mota-Fernandes D., Velez-Cruz R., Iltis I., Biard D., Egly J.M. (2010). NER factors are recruited to active promoters and facilitate chromatin modification for transcription in the absence of exogenous genotoxic attack. Mol. Cell.

[32] Adam S., Polo S.E., Almouzni G. (2013). Transcription recovery after DNA damage requires chromatin priming by the H3.3 histone chaperone HIRA. Cell.

[33] Polo S.E., Roche D., Almouzni G. (2006). New histone incorporation marks sites of UV repair in human cells. Cell.

[34] Oksenych V., Zhovmer A., Ziani S., Mari P.O., Eberova J., Nardo T., Stefanini M., Giglia-Mari G., Egly J.M., Coin F. (2013). Histone methyltransferase DOT1L drives recovery of gene expression after a genotoxic attack. PLoS Genet..

[35] Walmacq C., Cheung A.C., Kireeva M.L., Lubkowska L., Ye C., Gotte D., Strathern J.N., Carell T., Cramer P., Kashlev M. (2012). Mechanism of translesion transcription by RNA polymerase II and its role in cellular resistance to DNA damage. Mol. Cell.

[36] McKay B.C., Stubbert L.J., Fowler C.C., Smith J.M., Cardamore R.A., Spronck J.C. (2004). Regulation of ultraviolet light-induced gene expression by gene size. Proc. Natl. Acad. Sci. U.S.A..

