# Supplementary material for: DNA repair and recovery of RNA synthesis following exposure to ultraviolet light are delayed in long genes
Source: Nucleic Acids Res. 2015 Feb 26;43(5):2744–56. doi: 10.1093/nar/gkv148 (PMC4357734; doi:10.1093/nar/gkv148)
Supplement: SUPPLEMENTARY DATA [file supp_43_5_2744__index.html]

DNA repair and recovery of RNA synthesis following exposure to ultraviolet light are delayed in long genes — SUPPLEMENTARY DATA 

# DNA repair and recovery of RNA synthesis following exposure to ultraviolet light are delayed in long genes

## SUPPLEMENTARY DATA

**Files in this Data Supplement:**

- Supplemental Figures/Tables Legends
- Supplemental Figure 1
- Supplemental Figure 2
- Supplemental Figure 3
- Supplemental Figure 4
- Supplemental Figure 5
- Supplemental Figure 6
- Supplemental Table 1
- Supplemental Table 2
- Supplemental Table 3
- Supplemental Table 4
- Supplemental Table 5
