# Supplementary material for: DNA repair and recovery of RNA synthesis following exposure to ultraviolet light are delayed in long genes
Source: Nucleic Acids Res. 2015 Feb 26;43(5):2744–56. doi: 10.1093/nar/gkv148 (PMC4357734; doi:10.1093/nar/gkv148)
Supplement: SUPPLEMENTARY DATA [file supp_gkv148_nar-00185-d-2015-File009.docx]

SUPPLEMENTAL FIGURES/TABLES LEGENDS

**Supplemental Figure 1**. Transcription recovery following 10 J/m^2^ of UVC light in the *DRAM1*, *ANXA2* and *SESN1* genes. Direction of transcription is from left to right. (A) HF1 (B) XP-C, (C) CS-B and (D) HF1**,** irradiated with 20 J/m^2^.

**Supplemental Figure 2**. Enrichment of genes by functional pathways following irradiation with 10 J/m^2^ of UVC light in HF1 cells. Pathways are represented by genes induced or inhibited at least 2-fold following irradiation with UVC as assessed with Bru-seq. Pathways induced by A) 6 hours and B) 24 hours after irradiation. Pathways inhibited by C) 6 hours and D) 24 hours after irradiation. Enrichment was performed using the bioinformatics tool DAVID (david.abcc.ncifcrf.gov) using as background all the genes with RPKM > 0.3 in non-irradiated HF1 cells and the numbers shown represent the significance (p-value) of the enrichment value.

**Supplemental Figure 3.** Enrichment of genes by functional pathways following irradiation with 10 J/m^2^ of UVC light in XP-C cells. Pathways are represented by genes induced or inhibited at least 2-fold following irradiation with UVC as assessed with Bru-seq. Pathways induced by A) 6 hours and B) 24 hours after irradiation. Pathways inhibited by C) 6 hours and D) 24 hours after irradiation. Enrichment was performed using the bioinformatics tool DAVID (david.abcc.ncifcrf.gov) using as background all the genes with RPKM > 0.3 in non-irradiated HF1 cells and the numbers shown represent the significance (p-value) of the enrichment value.

Supplemental Figure 4. Enrichment of genes by functional pathways following irradiation with 10 J/m^2^ of UVC light in CS-B cells. Pathways are represented by genes induced or inhibited at least 2-fold following irradiation with UVC as assessed with Bru-seq. Pathways induced by A) 6 hours and B) 24 hours after irradiation. Pathways inhibited by C) 6 hours and D) 24 hours after irradiation. Enrichment was performed using the bioinformatics tool DAVID (david.abcc.ncifcrf.gov) using as background all the genes with RPKM > 0.3 in non-irradiated HF1 cells and the numbers shown represent the significance (p-value) of the enrichment value.

Supplemental Figure 5. Enrichment of genes by functional pathways following irradiation with 20 J/m^2^ of UVC light in HF1 cells. Pathways are represented by genes induced or inhibited at least 2-fold following irradiation with UVC as assessed with Bru-seq. Pathways induced by A) 6 hours and B) 24 hours after irradiation. Pathways inhibited by C) 6 hours and D) 24 hours after irradiation. Enrichment was performed using the bioinformatics tool DAVID (david.abcc.ncifcrf.gov) using as background all the genes with RPKM > 0.3 in non-irradiated HF1 cells and the numbers shown represent the significance (p-value) of the enrichment value.

Supplemental Figure 6. A) DNA lesion removal from both strands of the 5’-ends (TSS) and 3'-ends (END) of the genes *ATR, PAPPA, SLIT2* and a non-transcribed intergenic region. TC-NER deficient XP-C cells were irradiated with 10 J/m^2^ of UVC light and lesion removal estimated using quantitative long PCR. B) DNA lesion removal from both strands of *ANXA2*, *DRAM1* and the non-transcribing gene *LECT1 i*n XP-C cells as described in A). Note that lesions are repaired slower from the 3’-end compared to the 5’-end of long genes and similarly in the SLIT2 gene that showed no RNA synthesis recovery. Remaining DNA lesions are expressed as lesions/10 kb of the sense strand as calculated assuming a Poisson distribution (see Materials and Methods). The values represent the mean of quadruplicate samples from 2 independent biological experiments with bars representing the standard deviation. Sense strand normalization was related to DNA repair obtained from non-transcribed genes. Statistics with Two-way ANOVA: ”a” is significantly different from “b”.

**Supplemental Table 1.** Top 25 genes induced or inhibited following irradiation of HF1 cells with 10 J/m^2^ of UVC light. Induced genes 6 or 24 hours post UV are listed to the left and genes with inhibited transcription at 6 or 24 hours post UV are listed to the right, with gene name, size and the ratio compared to un-irradiated control cells.

**Supplemental Table 2.**  Top 25 genes induced or inhibited following irradiation of XP-C cells with 10 J/m^2^ of UVC light. Induced genes 6 or 24 hours post UV are listed to the left and genes with inhibited transcription at 6 or 24 hours post UV are listed to the right, with gene name, size and the ratio compared to un-irradiated control cells.

**Supplemental Table 3.**  Top 25 genes induced or inhibited following irradiation of CS-B cells with 10 J/m^2^ of UVC light. Induced genes 6 or 24 hours post UV are listed to the left and genes with inhibited transcription at 6 or 24 hours post UV are listed to the right, with gene name, size and the ratio compared to un-irradiated control cells.

**Supplemental Table 4.**  Top 25 genes induced or inhibited following irradiation of HF1 cells with 20 J/m^2^ of UVC light. Induced genes 6 or 24 hours post UV are listed to the left and genes with inhibited transcription at 6 or 24 hours post UV are listed to the right, with gene name, size and the ratio compared to un-irradiated control cells.

**Supplemental Table 5.**  Gene targets and primers used for quantitative long PCR.
