# Supplementary figures and images for: DNA repair and recovery of RNA synthesis following exposure to ultraviolet light are delayed in long genes
Source: Nucleic Acids Res. 2015 Feb 26;43(5):2744–56. doi: 10.1093/nar/gkv148 (PMC4357734; doi:10.1093/nar/gkv148)

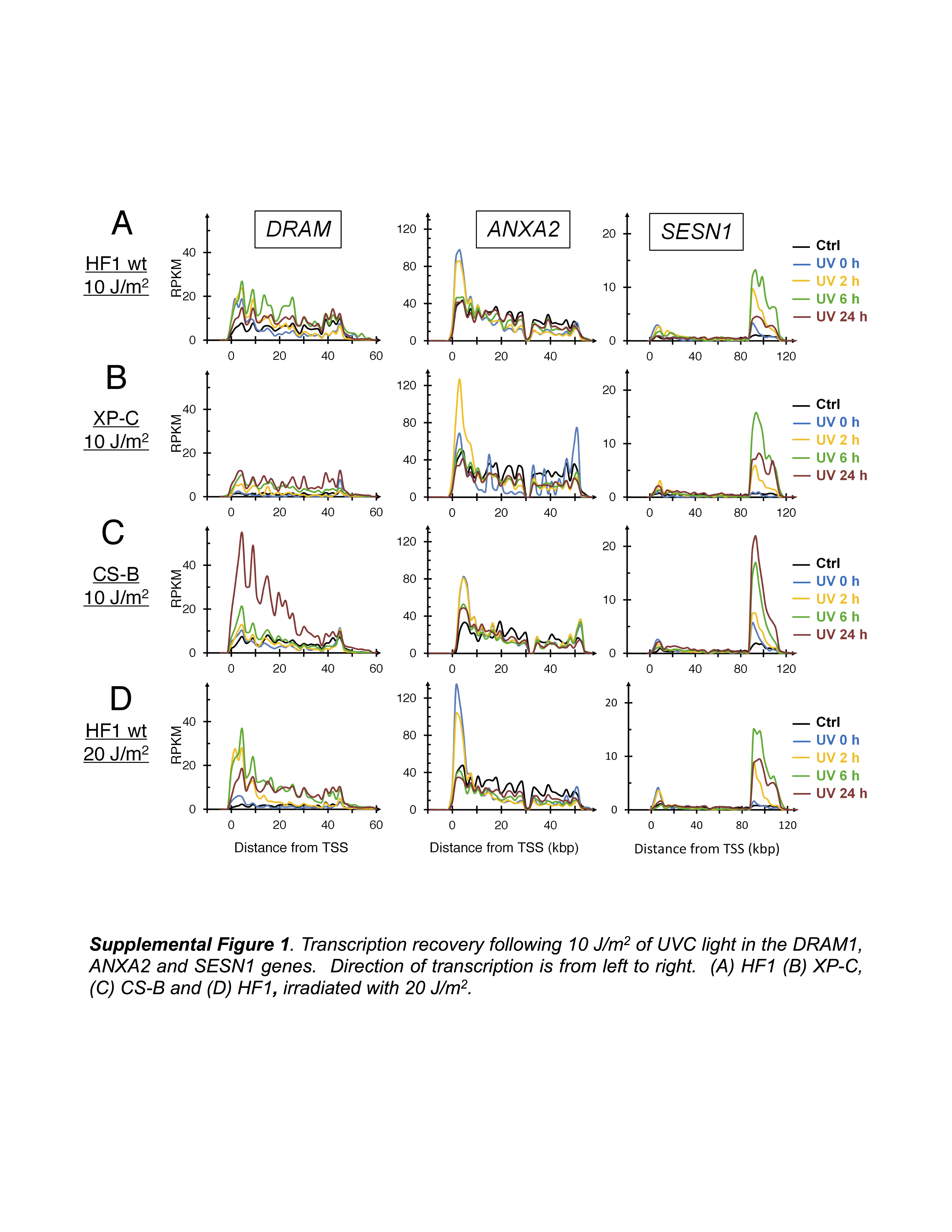

Supplement: SUPPLEMENTARY DATA [file supp_gkv148_nar-00185-d-2015-File010.png]

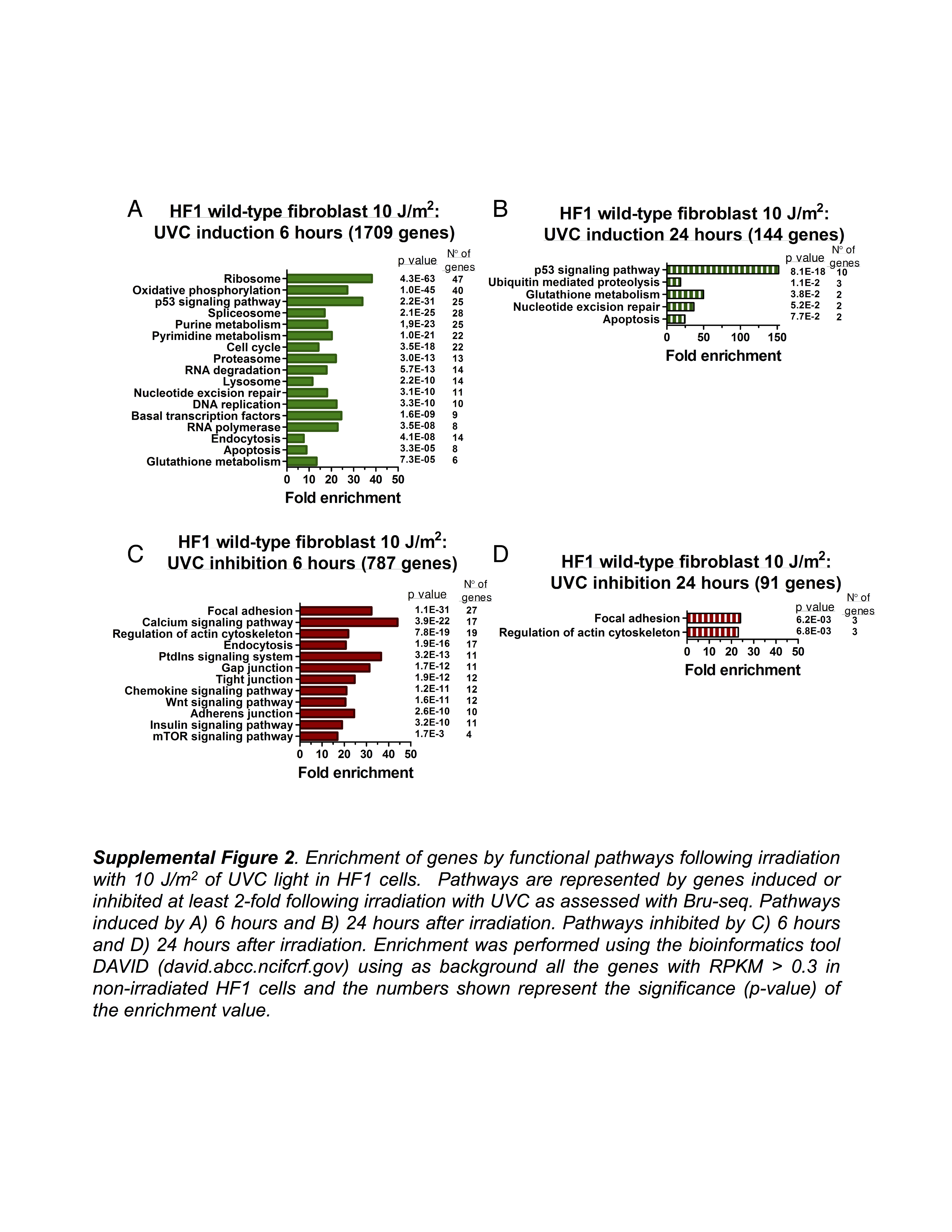

Supplement: SUPPLEMENTARY DATA [file supp_gkv148_nar-00185-d-2015-File011.png]

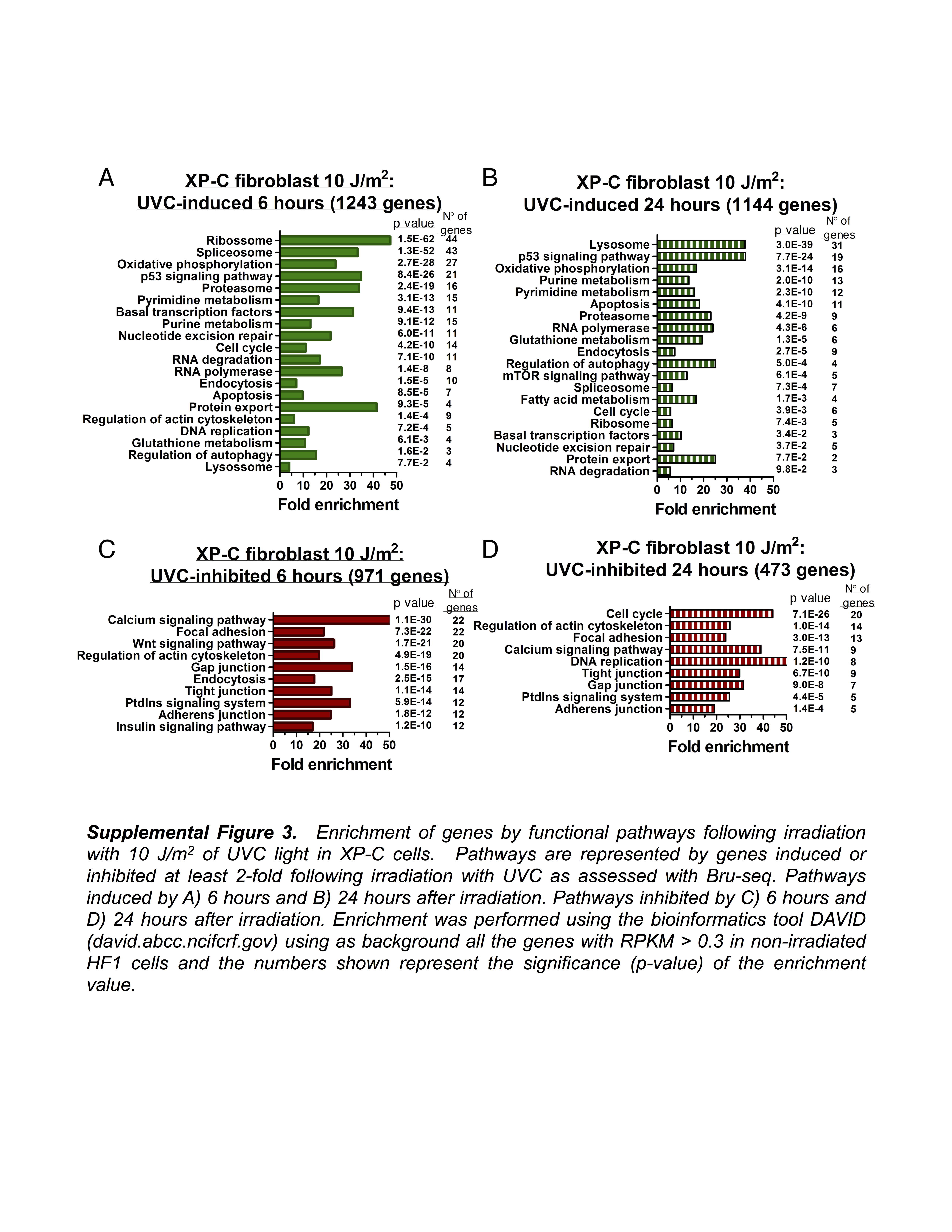

Supplement: SUPPLEMENTARY DATA [file supp_gkv148_nar-00185-d-2015-File012.png]

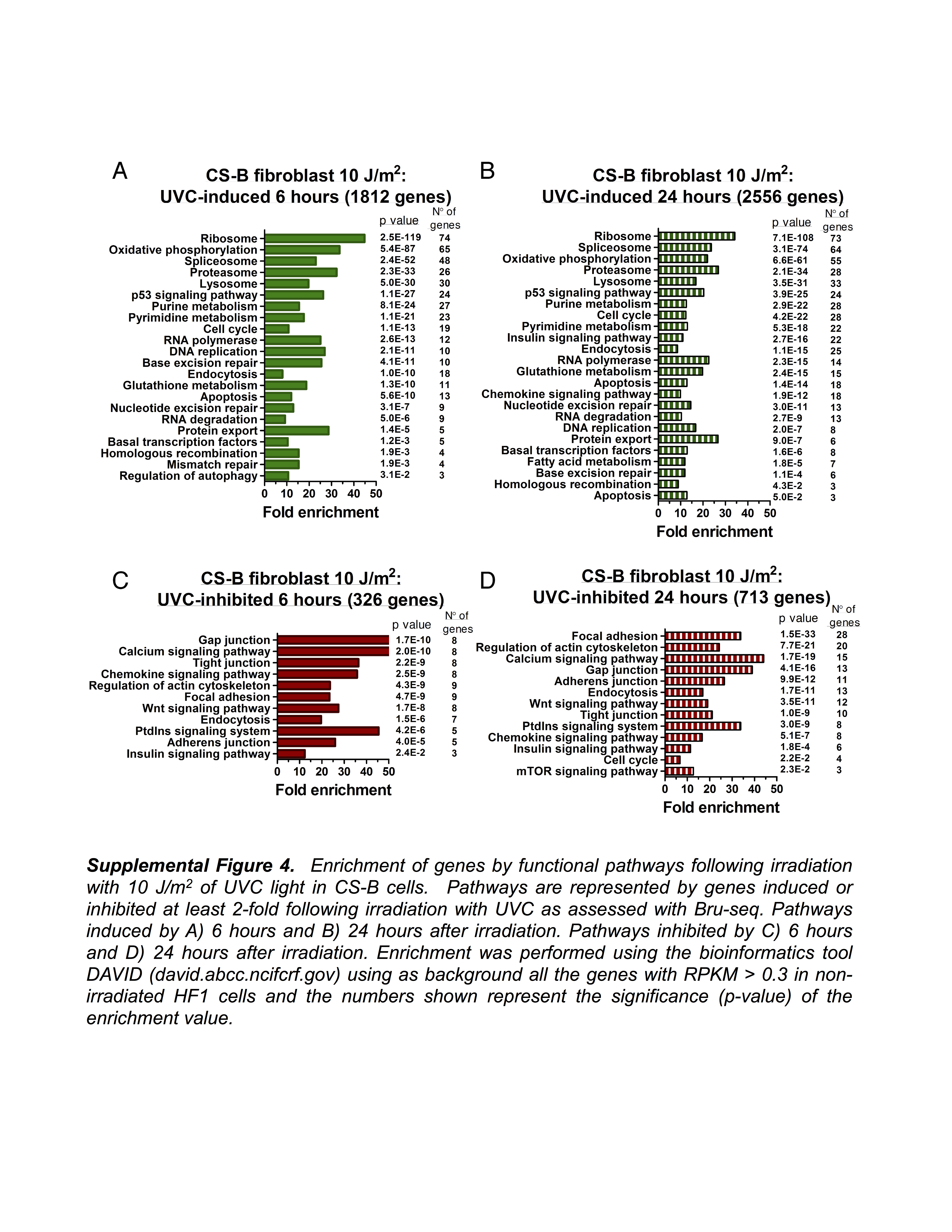

Supplement: SUPPLEMENTARY DATA [file supp_gkv148_nar-00185-d-2015-File013.png]

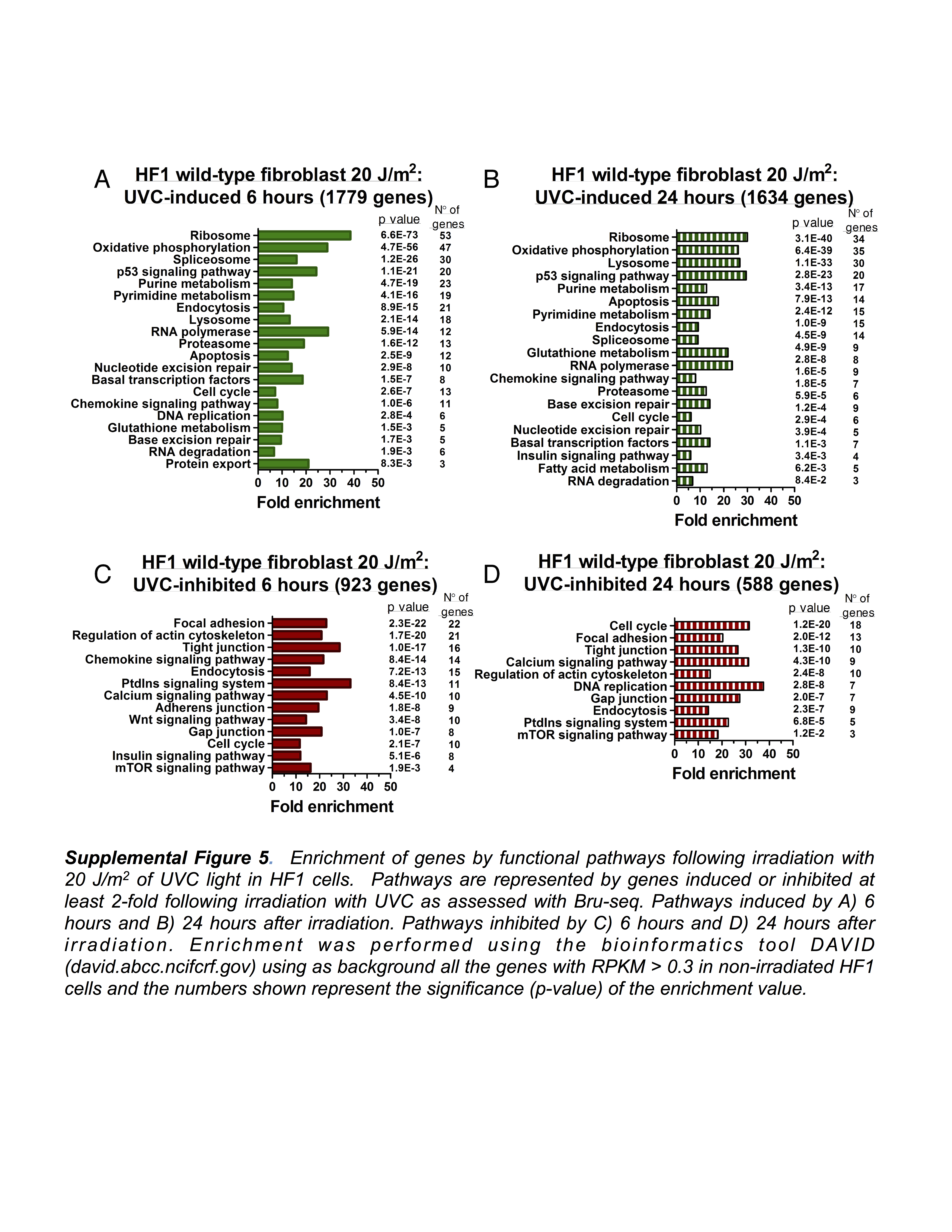

Supplement: SUPPLEMENTARY DATA [file supp_gkv148_nar-00185-d-2015-File014.png]

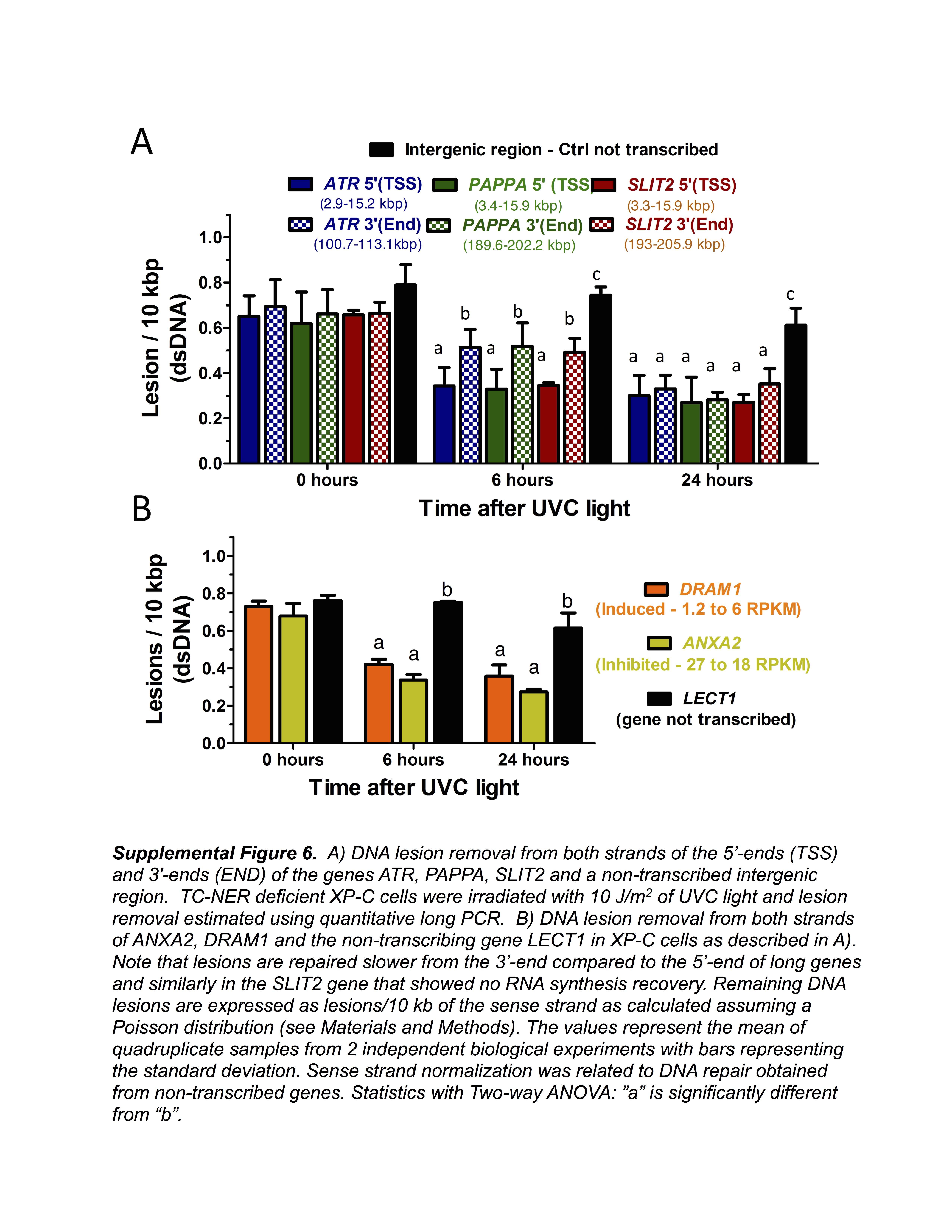

Supplement: SUPPLEMENTARY DATA [file supp_gkv148_nar-00185-d-2015-File015.png]

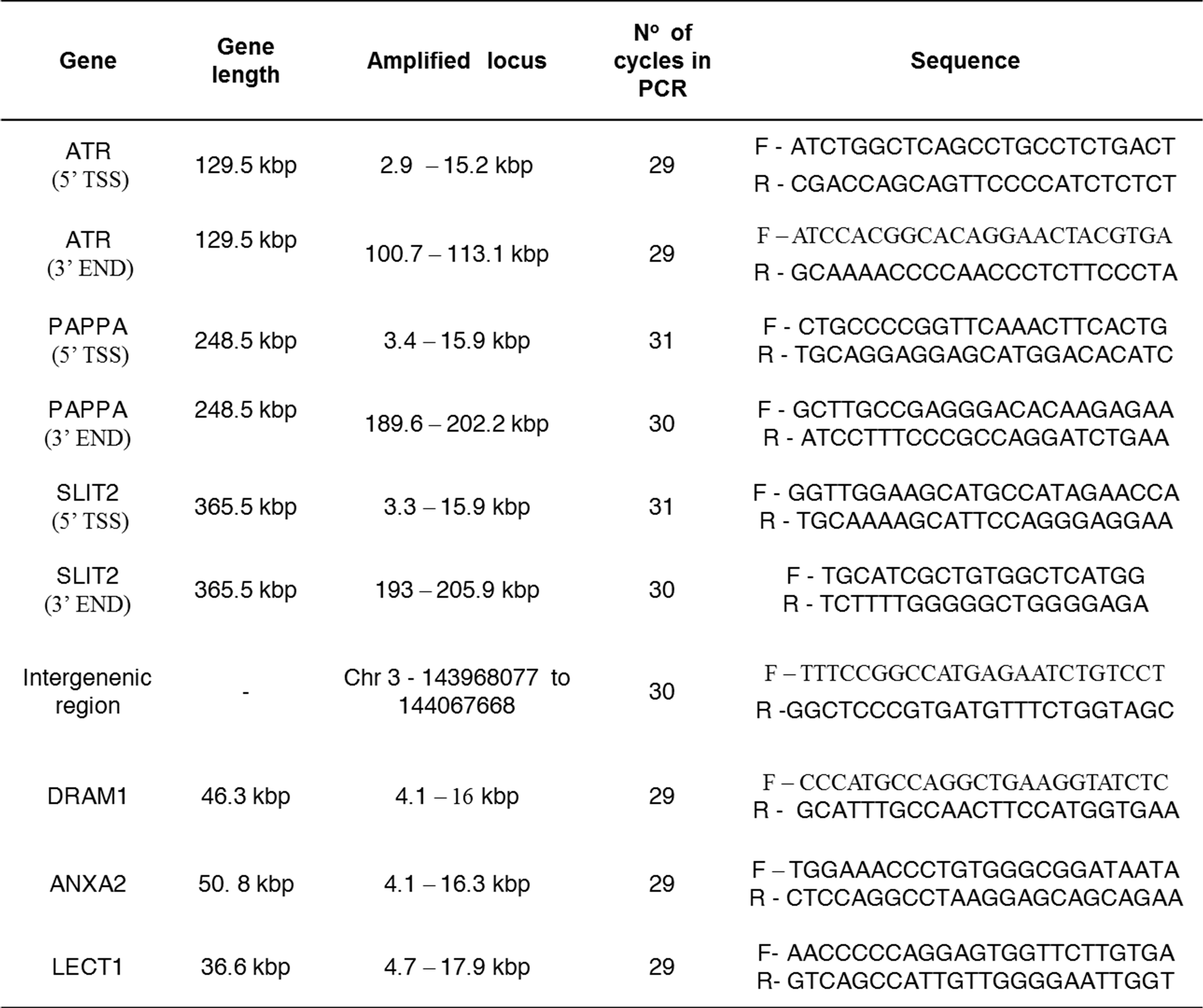

Supplement: SUPPLEMENTARY DATA [file supp_gkv148_nar-00185-d-2015-File020.png]
